# Molecular imaging of cardiac remodelling after myocardial infarction

**DOI:** 10.1007/s00395-018-0668-z

**Published:** 2018-01-17

**Authors:** Daniel Curley, Begoña Lavin Plaza, Ajay M. Shah, René M. Botnar

**Affiliations:** 1 GKT School of Medicine, King’s College London, London, UK; 2 School of Biomedical Engineering and Imaging Sciences, King’s College London, St. Thomas Hospital, 4th Floor, Lambeth Wing, London, SE1 7EH UK; 3 School of Cardiovascular Medicine and Sciences, King’s College London, London, UK; 4 The British Heart Foundation Centre of Excellence, King’s College London, London, UK; 5 Escuela de Ingeniera, Pontificia Universidad Catolica de Chile, Santiago, Chile

**Keywords:** Myocardial infarction, Cardiac remodelling, Cardiovascular imaging, MRI

## Abstract

Myocardial infarction and subsequent heart failure is a major health burden associated with significant mortality and morbidity in western societies. The ability of cardiac tissue to recover after myocardial infarction is affected by numerous complex cellular and molecular pathways. Unbalance or failure of these pathways can lead to adverse remodelling of the heart and poor prognosis. Current clinical cardiac imaging modalities assess anatomy, perfusion, function, and viability of the myocardium, yet do not offer any insight into the specific molecular pathways involved in the repair process. Novel imaging techniques allow visualisation of these molecular processes and may have significant diagnostic and prognostic values, which could aid clinical management. Single photon-emission tomography, positron-emission tomography, and magnetic resonance imaging are used to visualise various aspects of these molecular processes. Imaging probes are usually attached to radioisotopes or paramagnetic nanoparticles to specifically target biological processes such as: apoptosis, necrosis, inflammation, angiogenesis, and scar formation. Although the results from preclinical studies are promising, translating this work to a clinical environment in a valuable and cost-effective way is extremely challenging. Extensive evaluation evidence of diagnostic and prognostic values in multi-centre clinical trials is still required.

## Introduction

Cardiovascular disease (CVD) is the most common cause of death worldwide with the 2013 Global Burden of Disease Study estimating that almost a third of all deaths globally are attributable to CVD [1]. CVD remains a large health burden reflected in its position at the forefront of clinical research.

Coronary heart disease carries significant morbidity and is the leading cause of death across all diseases of the circulatory system. Myocardial infarction (MI) is mainly caused by the rupture of an atherosclerotic plaque leading to a thrombus forming within the lumen of a coronary vessel, which in turn blocks the blood flow to distal myocardium [61]. Infarction leads to cardiac myocyte death and subsequent necrosis of the tissue in the infarcted area, attracting inflammatory cells that phagocytose dead cells and debris within the infarcted area [134]. Inflammation plays a crucial role in cardiac healing post-MI contributing to the initial repair of the infarct, with replacement of dead myocytes by scar tissue. However, in the longer term, it also contributes to changes in ventricular shape and function involving infarct expansion, thinning of the myocardium, ventricular dilatation, hypertrophy of the remote uninfarcted myocardium, and an overall decline in cardiac function. These changes are collectively known as adverse ventricular remodelling and are associated with an increased likelihood of heart failure and mortality [92, 145]. Therefore, stratification of individuals at high risk of adverse ventricular remodelling post-MI may be of diagnostic, therapeutic, and prognostic benefits [62].

Current clinical imaging techniques are usually classified into anatomical and functional imaging. Plain film X-ray, computed tomography (CT), ultrasound, and magnetic resonance imaging (MRI) are mainly focused on structural changes, whereas nuclear medicine scans such as single proton-emission computed tomography (SPECT) and positron-emission tomography (PET) aim to provide functional aspects.

Molecular imaging is a novel technique which aims to visualise pathological processes at a molecular and cellular levels. Initially, molecular imaging was used for pharmaceutical development; however, recent research has focused on the use of molecular imaging as a clinical tool to stratify those patients at risk of developing disease and to provide early diagnosis [103]. This non-invasive, safe, and, therefore, attractive alternative to other invasive approaches such as tissue biopsy allows visualisation and measurement of underlying disease processes. Cardiac molecular imaging mainly involves imaging probes which are detectable using SPECT/PET and MRI, which are the focus of this review.

## Pathophysiology of post-MI cardiac remodelling

During MI, cardiomyocytes die as a result of oxygen deprivation due to blockage of a coronary artery which limits the blood supply to the cells, resulting in transmural ischaemia [18]. Under hypoxic conditions, cardiomyocytes undergo anaerobic respiration, destabilization of the cell membrane, and finally cell death [6, 73, 151]. Reperfusion after the acute event exacerbates existing oedema which gradually resolves as the myocardium repairs [158]. The infarct commences in the subendocardial layers in the centre of the area at risk, that is, the perfusion region of the coronary artery which has been occluded, and evolves towards the subpericardial layers and the boarder of the area at risk in a wavefront pattern if coronary occlusion persists [43, 109, 110].

Infarct size is a major indicator of post-MI remodelling, subsequent heart failure [44], and eventually prognosis [23, 94, 95]. It is determined by the size of the area at risk, the duration of coronary occlusion, and resulting ischaemia and the magnitude of collateral blood flow [54]. Temperature also impacts on the infarct size in the animal model [82] whereas the consensus on the haemodynamic situation, particularly heart rate [42], and myocardial oxygen demands have changed recently and it is now believed that they are only of limited importance regarding infarct size [43, 124].

The infarcted myocardium is morphologically characterised by myofibrillar contraction bands, swollen and ruptured mitochondria, destruction of cardiomyocyte membranes, microvascular destruction, haemorrhage, and inflammation [54]. These histological features reflect necrosis and become more apparent during reperfusion [60, 109, 110]. Necrotic cell death has many effects in infarcted myocardium [54], and different processes contribute such as excessive myofibrillar contractions [73, 104, 105, 136], digestion of the cytoskeleton and sarcolemma [56], and increased production of reactive oxygen species (ROS) [75, 118].

Contrary to necrosis, more regulated modes of cell death such as apoptosis, autophagy and necroptosis also occur in myocardial infarction, although their actual contribution to the final infarct size is still unclear [13, 54, 55, 64, 66, 72, 101, 146]. Apoptosis is an energy-dependent form of cell death with DNA disintegration and without an associated inflammatory response [7, 45, 71, 101, 154]. Autophagy is also a regulated mode of cell death characterised by lysosomal protein degradation and protein recycling, particularly mitochondrial proteins. Paradoxically, autophagy is considered to have a protective effect [107], although its role in human myocardial ischaemia is less well known [33, 122]. Necroptosis, as its name suggests, has similarities to both necrosis and apoptosis, but is distinctly regulated by activation of specific receptor-interacting protein kinases [100, 161].

After an acute MI, the most effective strategy for reducing the size of the infarct and improving clinical outcome is timely and successful myocardial reperfusion. However, the restoration of blood flow to the ischaemic myocardium can itself induce injury [8, 44, 124, 159]. In the last 30 years, many attempts have been evaluated to reduce the effects of reperfusion injury, processes known as pre-conditioning and post-conditioning [40, 141]. Whereby the myocardium is exposed to brief periods of ischaemia and reperfusion prior to (ischaemic pre-conditioning) [97, 124] or following an acute thrombotic MI (ischaemic post-conditioning) [124, 160].

The healing process after MI consists of inflammatory, proliferative, and maturation phases. The inflammatory phase involves the production of chemokines and cytokines which attract leucocytes to the infarcted zone. White blood cells such as neutrophils and macrophages phagocyte dead cells and extracellular matrix (ECM) debris. Then, during the proliferative phase, monocytes/macrophages contribute to tissue granulation by releasing cytokines and growth factors, suppressing inflammatory mediators and promoting angiogenesis, fibroblast growth, and production of ECM proteins. Finally, during the maturation phase, fibroblasts and vascular cells undergo apoptosis and a mature collagen scar is formed [15, 29, 145].

**Fig. 1:**
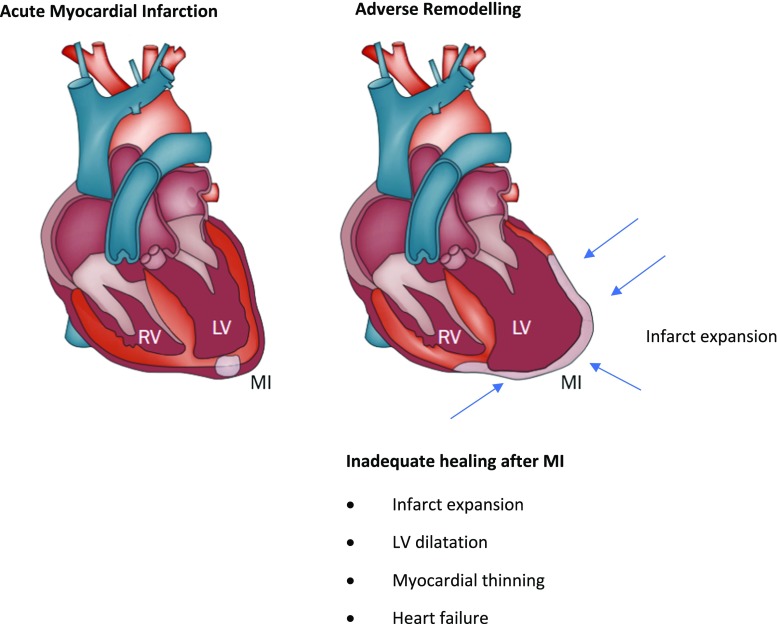
Schematic showing the gross changes in adverse cardiac remodelling post-MI (figure adapted from [143])

A further post-MI consequence is the stimulation of the renin–angiotensin–aldosterone system (RAAS), which leads to the activation of a family of proteolytic enzymes in the heart, named matrix metalloproteinases (MMPs), which are responsible for the degradation of extracellular proteins within the myocardium [85, 140]. In physiological conditions, MMPs are in the myocardium in an inactivated form. However, after MI, a significant decrease of tissue inhibitory MMPs (TIMPs) leads to MMPs’ activation [16, 156]. The main consequences of these cellular mechanisms include infarct expansion, left ventricular dilatation, and myocardial thinning, all of which contribute to heart failure (Fig. 1).

The size of the infarct zone and the level of perfusion, among other factors, affect the progression of these events. However, there is a direct correlation with early, aggressive immune/inflammatory responses associated with high concentrations of leucocytes and adverse remodelling leading to a poor prognosis [36, 63, 67, 106, 145]. Therefore, the ability to measure and visualise cardiac remodelling at the cellular and molecular levels may produce useful clinical information to tailor individual management plans for patients [62].

## Current cardiac imaging techniques

Almost all imaging modalities can be used to assess cardiac pathology. Although plain X-ray, CT, and ultrasound are currently used in clinical practice for cardiac imaging, these modalities are rarely used for molecular imaging. In this review, we will focus on SPECT, PET, and MRI, as they are the mainstay of molecular cardiac imaging.

## Single photon-emission computed tomography

SPECT imaging uses a gamma camera that rotates around the patient, sampling the radiation at various points to acquire a number of images which can then be reconstructed to produce a 3D image. Imaging of myocardial perfusion using SPECT is known as a rest/stress test. Clinically approved radioactive tracers such as thallium-201 (^201^Tl) and technetium-99 m (^99m^Tc) sestamibi or (^99m^Tc) tetrofosmin, are intravenously administered to the patient, taken up by cardiomyocytes that represent their initial distribution and this is seen as a marker for myocardial perfusion [142]. Image acquisition is performed while the patient is at rest and also while under stress, allowing the evaluation of myocardial viability and perfusion. Stress conditions can be achieved physically (e.g., exercise) or pharmacologically (e.g., adenosine or dobutamine) if the patient has poor exercise tolerance. Tomographic slices are then processed using iterative reconstruction with, e.g., a Weiner smoothing filter [80]. The final images are reconstructed in the short axis, vertical long axis, and horizontal long axis of the heart for both the resting and the stressed states and are quantified using a bull’s-eye plot [70].

SPECT is widely available and is clinically recommended for diagnostic and prognostic purposes for patients with suspected intermediate CVD [27]. However, its low spatial resolution and the use of ionising radiation represent a limitation, especially in patients who need repeated follow-up imaging.

## Positron-emission tomography

PET imaging differs slightly to SPECT, as the detectors are positioned in a stationary ring around the patient and PET tracers are biologically active allowing for assessment of, e.g., myocardial viability. Naturally occurring biological molecules can be radioactively labelled and administered to the patient. Clinical cardiac PET tracers include ^13^N-ammonia, ^15^O-water, and ^82^rubidium for myocardial perfusion and ^18^F-fluorodeoxyglucose (FDG) for cardiomyocyte metabolism and viability. The radioactive tracer decays, emitting a positron which travels in the tissue for a short distance before interacting with an electron, causing an annihilation event producing two 511 keV photons moving in opposite directions. The PET system detects these photons and, therefore, the localisation of the annihilation event. Correction for attenuation is a standard practice in PET imaging to improve accuracy and quantify concentrations of radioactive tracers [34]. A recent meta-analysis showed that sensitivity and specificity to detect obstructive CVD-induced ischaemia with PET imaging was 84–92%, while 81–85% was achieved using SPECT, demonstrating the higher diagnostic value of PET [59]. However, perhaps, the most important advantage of using PET compared to SPECT is its ability to use ^18^F-FDG to measure glucose metabolism within cardiomyocytes alongside myocardial perfusion. The combination of an ^18^F-FDG-PET metabolic scan together with a PET/SPECT perfusion scan enables to distinguish between infarcted and viable myocardial tissues [34].

## Cardiac magnetic resonance

Cardiac magnetic resonance (CMR) imaging is a non-ionising imaging modality, where patients are placed in a large magnetic field. Hydrogen atoms inside the patient align with the magnetic field and are perturbed by short radiofrequency (RF) pulses to generate an MR signal that can be spatially encoded with the help of strong magnetic field gradients. The combination of RF pulses and magnetic field gradients is known as a pulse sequence. After perturbation (also called excitation), the precessing hydrogen atoms emit a signal that can be measured with a receiver coil and spatially encoded in the presence of magnetic field gradients. Subsequent reconstruction of the MR signal which acquired in a 2D or 3D space, also referred to k-space, typically by a Fourier transform, reveals spatially resolved information about the structure being imaged [86]. CMR is a well-established cardiac imaging technique that allows the assessment of the anatomy and function of cardiac tissue through visualisation of cardiac tissues due to differences in T1 and T2 relaxation time and blood flow and by employing cine imaging [91, 147, 152]. In addition, CMR can identify the area at risk after MI due to the oedematous nature of this region, as T1-weighted and T2-weighted images are both sensitive to water content (long T1 and long T2) [30, 47]. However, the use of T2-weighted MRI together with LGE quantification to assess oedema has been criticized due to the spatial and temporal dynamics of the oedema after reperfusion. In addition, motion artefacts and/or artificial hyperintensities could affect the quantification of oedema [46]. Furthermore, the use of contrast agents significantly improves the detection and evaluation of injured areas. Gadolinium-based contrast agents can be monitored on their first-pass to assess cardiac perfusion; areas that are poorly perfused will have reduced signal intensity on T1-weighted images as less gadolinium will be present in this area [58]. Gadolinium-based contrast agents can also be used to determine areas of irreversible damage, as it clears from necrotic and fibrotic tissue much slower than healthy tissue [14] and thus leads to a late gadolinium enhancement (LGE) effect.

## Molecular imaging techniques in post-MI remodelling

Molecular imaging is a non-invasive imaging technique to detect biological processes in vivo. This is achieved with the use of tracers that bind to specific biological molecules that can be visualised by the imaging system [103].

Methods involved in nuclear molecular imaging to visualise post-MI cardiac remodelling are similar to the standard nuclear medicine techniques previously discussed, whereby radioactive molecules are taken up by cardiomyocytes to visualise myocardial perfusion. However, novel techniques now use radioactively labelled tracers that target specific molecules involved in the cellular process of cardiac remodelling.

Conversely, CMR has traditionally been used for visualisation of whole organ anatomy, function, perfusion, and fibrosis, as described above. However, a recent shift within the literature and advancement of nanotechnology and new imaging probes has enabled molecular imaging of specific targets using MRI. Imaging agents have been developed to visualise specific targets involved in the cellular and molecular pathways during post-MI remodelling. Gadolinium chelates have been used as extracellular MRI contrast agents for years. More recently, gadolinium chelates have been successfully employed for imaging highly abundant targets such as albumin, fibrin, collagen, and elastin.

## Extracellular matrix

**Fig. 2:**
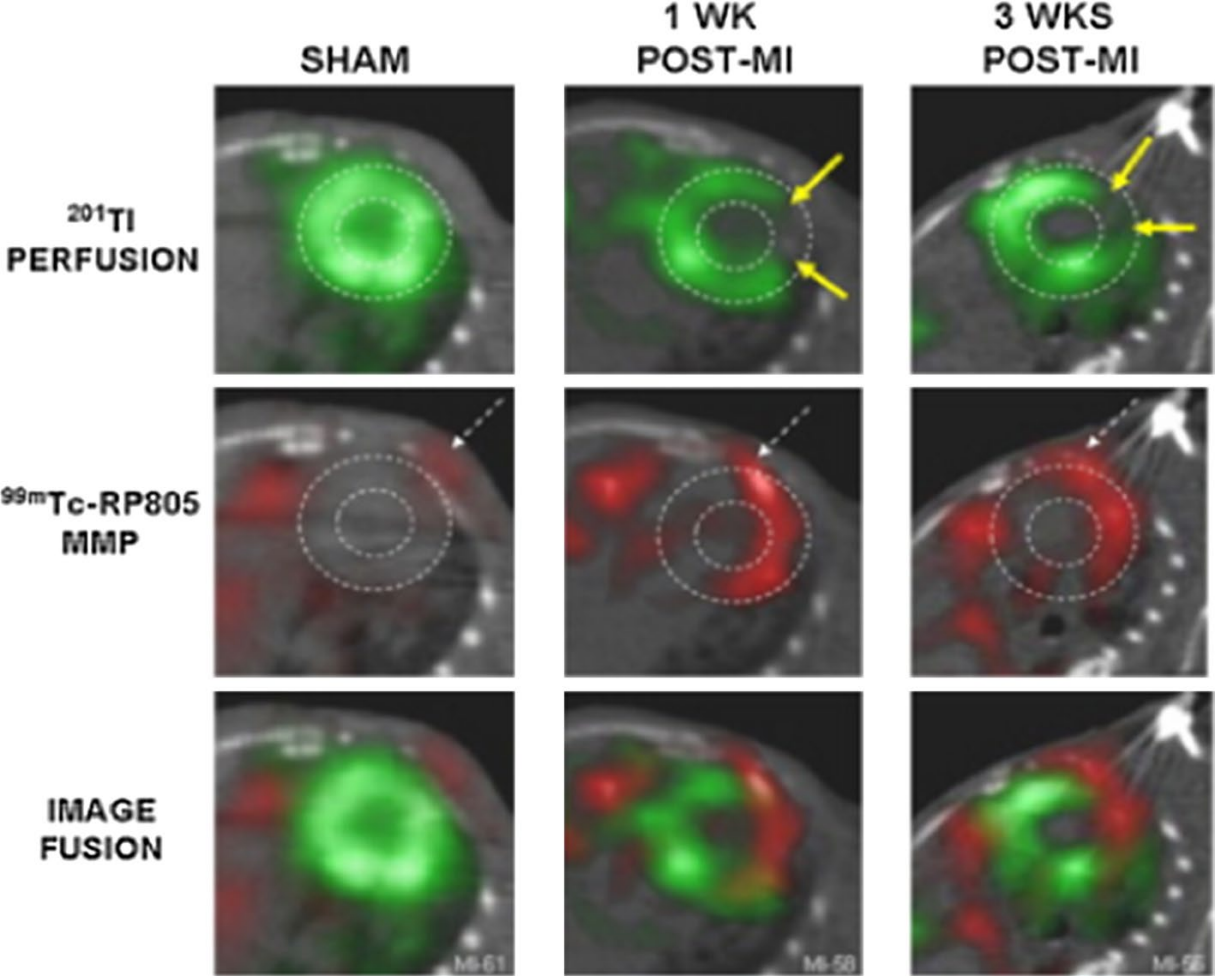
Thalium-201 perfusion imaging, Tc-99m-labelled MMP imaging, and fused images before (SHAM), 1 week and 3 week post-MI using SPECT/CT. Arrows show infarct zone, which is poorly perfused, where MMPs are detected [132]

An interesting target to evaluate post-MI alterations is the activation of MMPs, in particular MMP-2 and MMP-9, as they are involved in ECM degradation and cardiac remodelling post-MI [17, 131, 155]. It has been shown that radiolabelling molecules that target MMPs allow visualisation of activated MMP post-MI in vivo. Su et al. [132] showed in a murine model of MI that radiolabelled MMPs can be visualised using SPECT/CT in areas of infarction, although there is some signal within non-ischaemic areas of the heart, demonstrating the global MMP activation and remodelling (Fig. 2). This study, like many others, suggests that activation of MMPs occurs mainly in areas of infarction and highlights the potential for evaluation of ventricular remodelling.

**Fig. 3:**
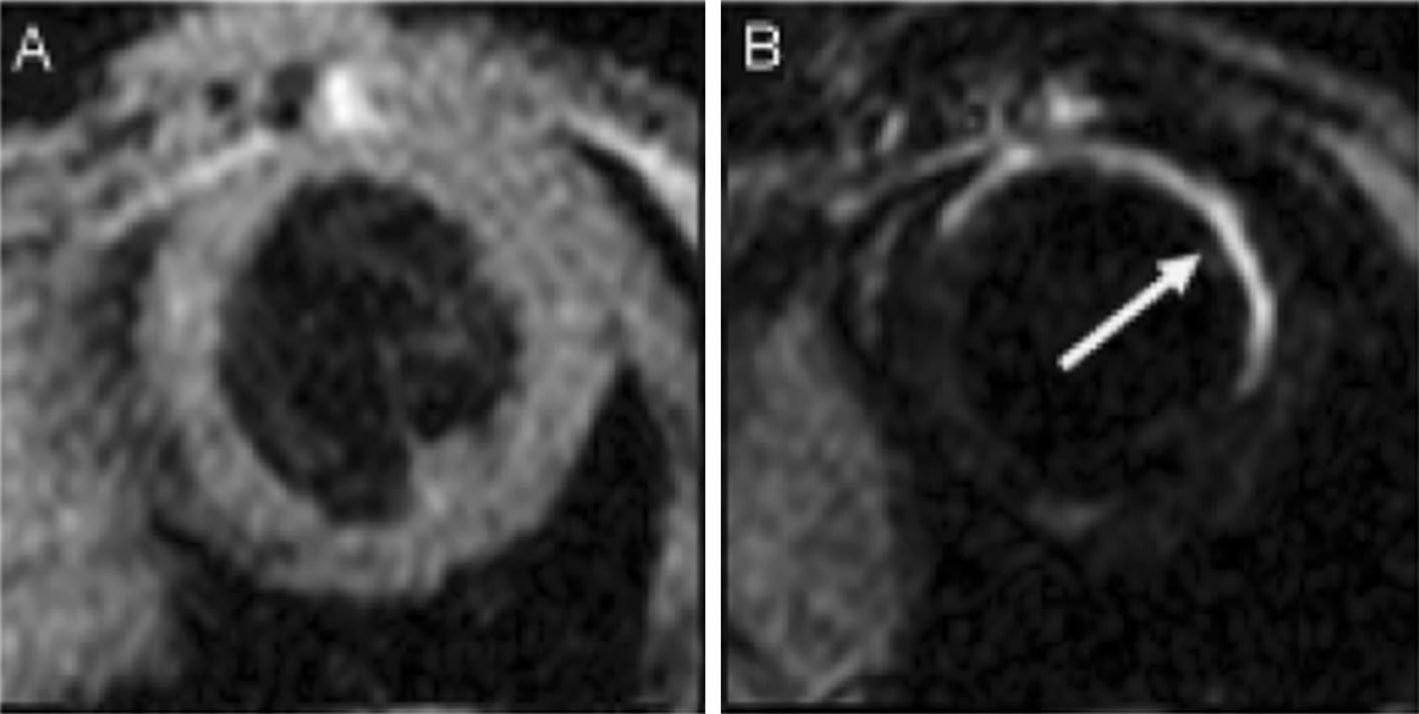
T2-weighted CMR image before injection of EP-3533 (**a**) and **b** an inversion recovery CMR image 40 min after the injection of EP-3533. The arrow highlights the hyper-intense region indicating high levels of collagen, therefore, scar [11]

In addition, collagen has been targeted using CMR. EP-3533, a gadolinium-based contrast agent, has been studied in mouse models of MI [11, 41]. This imaging probe is of small molecular weight and was developed to visualise the collagen within post-MI scar. This differs slightly from the standard gadolinium chelates that can visualise the scar through gross changes within the cardiac tissue. Hyper-intensity was seen, 10 min after injection of EP-3533, and its washout times were significantly longer than that of Gd-DTPA in areas of scar and in normal myocardium. Therefore, EP-3533 is able to image fibrosis in a mouse model of post-MI scarring (Fig. 3).

## Renin–angiotensin–aldosterone system

In addition to the ECM, the renin–angiotensin–aldosterone system (RAAS) has also been proposed as a possible target. Various factors may activate the RAAS such as a loss of blood volume or a drop in blood pressure (as in haemorrhage or dehydration). Local cardiac levels of molecules involved in the RAAS are increased in post-MI states, being potential targets for imaging ventricular remodelling post-MI [102]. Owing to the pivotal role that angiotensin-converting enzyme (ACE) inhibitors play in CVD treatment, ACE inhibitor-based tracers are an attractive imaging approach to monitor disease progression and therapeutic interventions. Thus, several targeted radioactively labelled pharmaceuticals have been developed [120]. ^18^F-captopril [53] and ^18^F-flurobenzoyl-lisinopril [79], two radiolabelled ACE inhibitors, have shown increased levels in the infarcted area using PET imaging [20]. Lisinopril has also been successfully labelled with ^99m^Tc in rats [24, 25] and it is thought that it has higher affinity for tissue ACE than captopril, as shown in an experimental in vitro study [133]. These tracers allow distribution assessment while maintaining the therapeutic inhibition of ACE with angiotensin II type-1 receptors (AT_1_R) in vivo [19, 121].

A PET tracer ^11^C-zofenoprilat (a derivative of the ACE inhibitor zofenopril) has also been described and evaluated in humans; however, it accumulates mainly in tissues with high levels of ACE, such as the liver, lungs, kidneys, and gallbladder. Therefore, the use of this tracer in cardiac imaging is of little interest [88].

Other authors have described the use of AT_1_R as imaging targets for heart failure and LV remodelling. Radiolabelled tracers include ^11^C-MK-996, ^11^C-L-155884, SK-1080, and ^11^C-KR31173 an analogue of SK-1080 [35, 77, 89, 90, 135]. In addition, uptake of losartan, an angiotensin receptor blocker labelled with ^99m^Tc for SPECT imaging, has been shown to increase by 2.4-fold in post-MI mouse models when compared to controls [150].

The PET agent ^11^C-KR31173 has been effective in a rat MI model showing a peak uptake in the infarct zone at 1–3 week post-surgery. This effect can be blocked entirely using the AT_1_R antagonist valsartan, in comparison with the ACE inhibitor enalapril which did not affect AT_1_R density, providing a platform to predict the risk for ventricular remodelling and to monitor the efficacy of anti-RAAS drug therapy [48]. Furthermore, healthy pig studies of ^11^C-KR31173 confirmed myocardial uptake with regional homogeneousness and AT_1_R specificity with the use of blocking experiments. This study included the first human trial in which there were no adverse effects across all subjects (*n* = 4). The results of the human studies showed detectable and specific myocardial retention of ^11^C-KR31173, though at a lower level than pigs. Myocardial retention disappeared after blockage with olmesartan, an AT_1_R antagonist, demonstrating its affinity for the AT_1_R [31]. Inter-species differences have been reported within the literature with rats [48] and mice [150] showing strong upregulation of AT_1_R in infarcted myocardium in contrast to pigs, where this is less pronounced [31]. Human subjects show significantly lower levels of absolute retention of AT_1_R than pigs. Whether this is due to further inter-species differences or the effects of anaesthesia in animals has not yet been clarified [31]. The potential for imaging the AT_1_R remains an exciting prospect, especially given the recent use of ^11^C-KR31173 in humans indicating the potential safety of the tracer which will require further evaluation in clinical trials.

## Angiogenesis

One of the most important biological processes during the proliferation phase of myocardial healing after ischaemic injury is microvascular angiogenesis, which consists of the development of new blood vessels from pre-existing vasculature [112]. Angiogenesis is stimulated by increased levels of vascular endothelial growth factor (VEGF) and basic fibroblast growth factor (bFGF), which are released in response to the infarction. Imaging targets of angiogenesis include α_v_β_3_ integrin [49] and VEGF receptors [115].

α_v_β_3_ integrin is essential for endothelial cell propagation and survival. It is generally not expressed on mature vessels in physiological conditions; however, it is expressed on endothelial cells during vasculogenesis and angiogenesis as a response to angiogenic growth factors [22]. Integrins recognise proteins and surface molecules through short peptide sequences such as Arg–Gly–Asp (RGD) [37]. Several studies have been done to explore tracers targeting α_v_β_3_ integrin in tumour models [49]. However, some studies have been focused on the evaluation of radiolabelled tracers targeting α_v_β_3_ integrin in cardiac angiogenesis, including ^18^F-galacto-RGD and ^99m^Tc-RAFT-RGD [21].

^18^F-galacto-RGD is a PET tracer that binds α_v_β_3_ integrin developed by Haubner et al. [38, 39]. Higuchi et al. [49] demonstrated in rats that ^18^F-galacto-RGD levels, rise 3 days, peak around 3 weeks, and return to baseline levels 6 months after MI. Makowski et al. [84] concurred and showed that ^18^F-galacto-RGD levels are raised in patients 2 weeks after MI. Furthermore, correlation between early post-MI uptake of this tracer and the absence of significant LV remodelling after 12 weeks follow-up has been demonstrated by Sherif et al. [119].

However, the production of ^18^F-galacto-RGD is challenging due to the multistep synthesis and the need for an on-site cyclotron. Therefore, alternative RGD tracers have been proposed such as the one-step labelled PET tracer ^18^F-AlF-NOTA-PRGD2 [32] which shows a similar pattern of tracer uptake in the infarct area and significantly higher tracer levels than those reported using ^18^F-galacto-RGD [49]. The in vivo performance and easy production method of this PET tracer may facilitate its future clinical translation. It has also been used successfully to visualise angiogenesis after VEGF gene therapy and bone-marrow stem-cell therapy in rats [10].

A further two gallium-based tracers have been studied, again to offer an alternative for the challenges faced by the production of ^18^F-galacto-RGD, as gallium tracers may be beneficial to sites which do not have a cyclotron close by. Laitenen et al. have shown both ^68^Ga-NODAGA-RGD and ^68^Ga-TRAP(RGD)_3_ to be as effective as ^18^F-galacto-RGD in a rat model [74]. Although the prime importance of these tracers is the imaging quality, these more practical aspects of introducing these tracers into clinical practice will be a decisive factor into deciding which of these tracers to take forward into clinical trials and ultimately translate to the bed side.

**Fig. 4:**
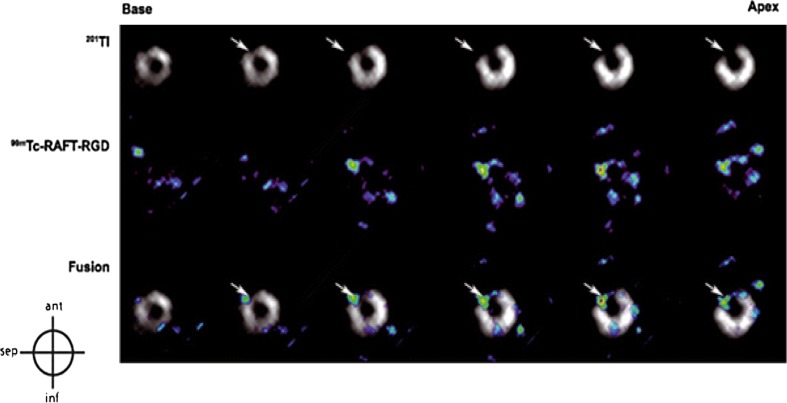
Myocardial short-axis images from base to apex with ^201^Tl perfusion SPECT, ^99m^Tc-RAFT-RGD SPECT scan and fused images. The arrows highlight areas of infarct (figure adapted from [21])

^99m^Tc-RAFT-RGD is an SPECT tracer that also binds α_v_β_3_ integrin and has been validated to image myocardial angiogenesis on rat models in vivo [21]. Figure 4 highlights the ability of this tracer to identify areas of active angiogenesis when compared with ^201^Tl perfusion scans that identify areas of ischaemia. These results showed the maximum quantitative uptake in the infarct area at 2 weeks after MI which is comparable to Higuchi et al. [49] using the PET tracer ^18^F-galacto-RGD 1 or 3 weeks following reperfusion in a similar murine model.

Meoli et al. have evaluated an SPECT tracer ^111^In-RP748, which shows a similar increase in tracer activity to that of ^99m^Tc-RAFT-RGD in the re-perfused zone post-MI [93]. The authors also showed an infarct-to-normal zone tracer activity ratio of 1–1.6, with dual isotope SPECT imaging of ^111^In-RP748 and ^99m^Tc-MIBI in a canine model.

CMR imaging with α_v_β_3_-targeted paramagnetic nanoparticles is currently an active area of research, and although this is not yet used to assess post-MI remodelling, it has been studied in the context of atherosclerosis [9, 157].

All of the above tracers are aimed at targeting the α_v_β_3_ integrin signal which is actually a rather controversial topic. This is owing to the signal may not solely represent angiogenesis, but also myofibroblast and leucocyte activity, although more studies need to be performed to evaluate these effects [4, 50, 143, 144]. Furthermore, the post-MI uptake of an SPECT tracer targeting α_v_β_3_/β_5_ has been shown to predict the extent of fibrosis 1 year later, which highlights the potential of this signal to be used to visualise myofibroblasts [149].

VEGF is an abundant and potent angiogenic agent and its receptors are potentially good targets for imaging of angiogenesis. ^111^In-labelled recombinant human VEGF_121_ was used to visualise areas of active angiogenesis in a rabbit model with unilateral hind limb ischaemia. In this study, tracer levels were detected using scintillation well counting and planar scintigraphy studies, demonstrating that the tracer uptake in ischaemic muscle was significantly increased, 10 days after occlusion [81]. More recently, ^64^CU-DOTA-VEGF_121_ has been explored as a PET tracer, targeting VEGF receptors in a rat model of MI. In this study, tracer levels peaked 3 days post-MI and decreased over time until it reached baseline levels on day 24 [115].

There are clearly many tracers, predominantly radiolabelled PET and SPECT tracers that have been evaluated in animal models to visualise angiogenesis for the purpose of post-MI remodelling, with some having even been tested on humans. There is a growing consensus that this technology could be of pronounced clinical benefit to define the risk of patients who may develop cardiac remodelling post-MI. There is, therefore, a need for further evaluation of these tracers in clinical trials. However, many challenges, as discussed through this section, have been identified in bringing these tracers to the bedside and researchers will have to choose which tracer to invest in for their studies. Furthermore, the specificity of the α_v_β_3_ integrin to angiogenesis has been questioned which should play a role in future studies.

## Apoptosis

Apoptosis can also be evaluated using imaging techniques by targeting the protein annexin V which is expressed on the cell surface of apoptotic cells [51]. Kietselaer et al. [66] successfully labelled annexin V to ^99m^Tc, allowing for visualisation of apoptosis using SPECT, showing a direct correlation between annexin V uptake and deterioration in left ventricular function.

**Fig. 5:**
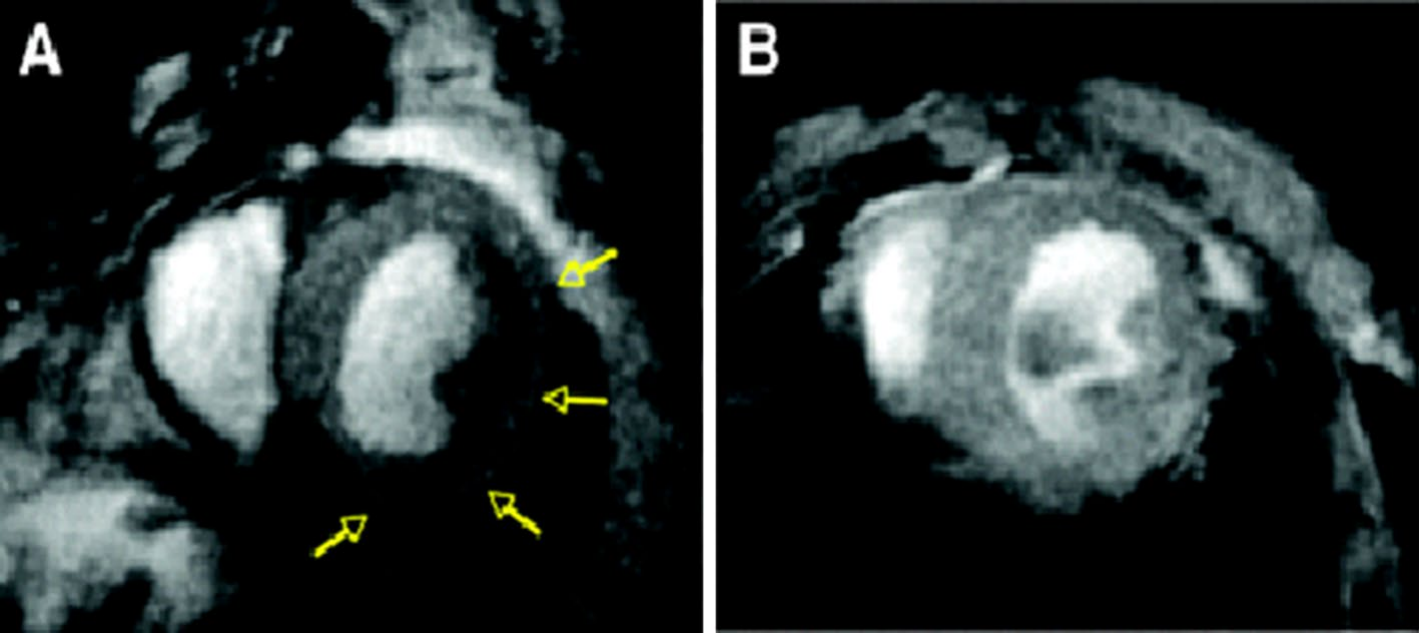
Post-MI state in mouse models injected with AnxCLIO-Cy5.5 (**a**) and a control probe inact_CLIO-Cy5.5 (**b**). Significant hypo-intensity can be seen with AnxCLIO-Cy5.5 (**a**), depicted by yellow arrows. There are no areas of significant uptake seen using the control probe (**b**). Regions of hypo-intensity represent the visualisation of active apoptosis [128]

First, MRI approaches to image apoptosis were reported by Sosnovik et al. [127] using AnxCLIO-Cy5.5, a novel annexin-based magneto-optical nanoparticle. They reported a significant decrease in myocardial T2* compared to the unlabelled control probe (Fig. 5). In addition, a significant correlation was reported between the local extent of signal loss and the infarcted area, suggesting that the AnxCLIOCy5.5 probe accumulated specifically in regions of injured and apoptotic myocardium. More recently, Sosnovik et al. [128] presented a dual contrast-molecular MRI approach to simultaneously evaluate apoptosis and necrosis. In this study, AnxCLIOCy5.5 was used to image apoptosis and a gadolinium chelate, Gd-DTPA-NBD, was used to detect cardiac necrosis. Interestingly, only 21% of the myocardium with active apoptosis colocalizes with the Gd-DTPA-NBA signal, suggesting that viable myocardium may be present within the apoptotic area.

## Inflammation

During ischaemic injury, the infarct area becomes oedematous due to increased capillary permeability and macrophages migrate to the infarct zone, where they accumulate. Magnetic iron oxide nanoparticles (MNPs) are ideal for imaging scarce molecular targets within cardiac tissue [130], as they are very small (nanometres), have high magnetic relaxation properties, and are designed to be biologically inert [126]. During the inflammatory phase, MNPs extravasate into the infarcted myocardium, permitting them to accumulate at the imaging target. MNPs are recognised as foreign bodies and are taken up by phagocytes in the infarct zone and, therefore, can be used to visualise inflammation within the myocardium. A T2*-weighted gradient-echo sequence is performed and MNP accumulation is seen as hypo-intense regions within the image, due to the high relaxivity of MNPs.

An additional probe to image the inflammatory phase is the use of perfluorocarbon nanoemulsions (^19^F). These particles are avidly taken up by macrophages which then migrate to the infarcted zone. Low-resolution ^19^F-MRI has been validated to visualise macrophages in a post-MI state. Macrophage accumulation was detected within the infarcted area over time [26].

An additional approach to image inflammation is targeting myeloperoxidase (MPO), an enzyme produced by neutrophils and monocytes that has been correlated with an adverse effect on LV remodelling and function [148]. Gadolinium-labelled MPO has been validated for assessment of MPO activity in post-MI myocardium with a significant increase 2 days after myocardial injury [98].

**Fig. 6:**
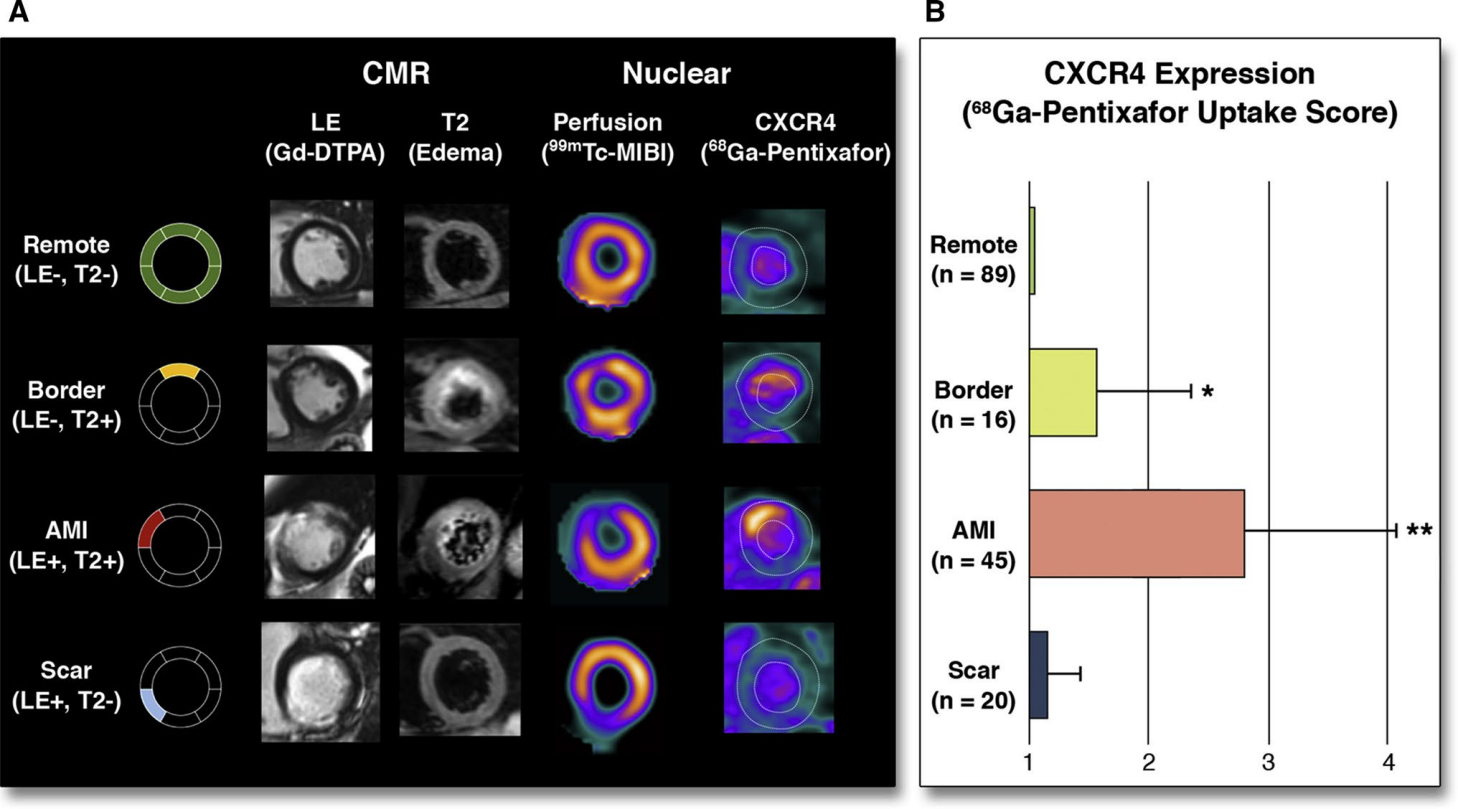
Uptake of 68 Ga pentixafor in patients after acute ST-segment elevation myocardial infarction indicating various levels of CXCR4 expression in myocardial segments with different patterns of myocardial injury as defined by the presence (+) or absence (−) of gadolinium-diethylenetriamine pentaacetic acid (DTPA) late enhancement (LE) or edema on T2-sequences (T2) at cardiac magnetic resonance imaging. **a** Representative short-axis slices characterizing four different types of segments. **b** Results of segmental pentixafor uptake score in respective segment types. *p < 0.05 versus remote; **p < 0.05 versus all others. *HLA* horizontal long axis, *SA* short axis [139]

Several preclinical studies have shown how ^18^F-FDG-PET can be used to evaluate the innate immune response after MI [57, 78, 116]. Using a similar approach ^18^F-FDG-PET uptake has been successfully correlated inversely with the functional outcome 6 month post-MI [114], presenting ^18^F-FDG-PET uptake as a possible marker of myocardial outcome. However, imaging of inflammation using ^18^F-FDG has few limitations. First, ^18^F-FDG is a glucose analogue and can be used for the metabolism of different cells. Moreover, ^18^F-FDG is generally used to evaluate myocardial viability [117] and metabolism response to hypoxia [2]. Therefore, imaging inflammation using ^18^F-FDG in the heart requires the suppression of cardiomyocytes by dietary pre-preparation of the patients [138]. However, it has not been shown how reliable this method is to suppress signal coming from viable cardiomyocytes after MI or hibernating myocardium, among others. Other limitations of the use of ^18^F-FDG are that it is not possible to differentiate between different subpopulations of inflammatory cells, which play different roles in myocardial healing. Therefore, it is crucial to develop new targeted tracers to detect the different subpopulations of inflammatory cells. In light of that, it has been proven in cells, animals and men that the tracer ^11^C-Methionine is taken up by inflammatory cells, preferentially inflammatory macrophages [137]. The absence of cardiomyocyte uptake renders ^11^C-methionine as a very attractive tracer for imaging inflammation post-MI. Finally, PET imaging with ^68^Ga-pentixafor targeting CXCR4, a protein involved in leukocyte recruitment to the injured region, has shown robust results in the infarcted myocardium in mice (Fig. 6) [139]. The use of this tracer in a small cohort of patients has shown more heterogeneous results, not providing any correlation between tracer uptake and any clinical predictive parameter. However, further larger and controlled cohort studies testing the usefulness of ^68^Ga-pentixafor imaging to determine outcome post-MI are required.

## Magnetic resonance spectroscopy

**Fig. 7:**
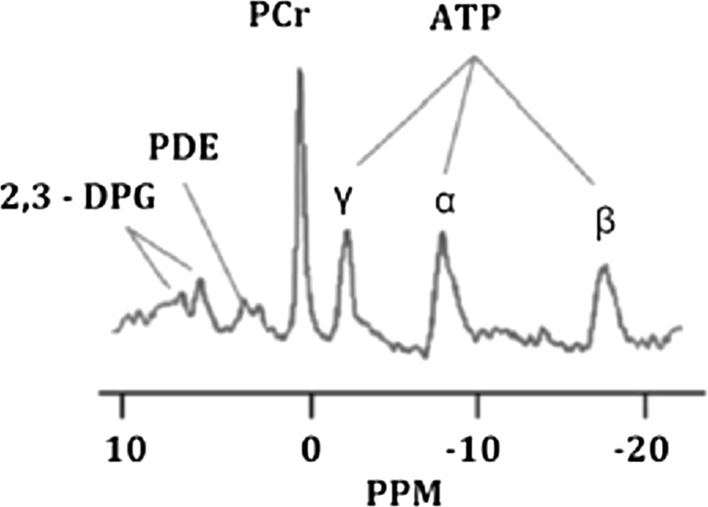
Typical ^31^P-magnetic resonance spectroscopy spectrum showing 2,3-diphosphoglycerate (2,3-DGP), phosphodiester (PDE), phosphocreatine (PCr), and the three phosphorus peaks of ATP (γ, α, and β). The *x*-axis is expressed in parts per million (ppm) [108]

Magnetic resonance spectroscopy (MRS) is the only technique that allows the evaluation of metabolites in the myocardium without the use of external contrast agents in vivo. MRS uses similar acquisition principles to MRI; however, it requires special broad band RF amplifiers and multinuclear RF coils to evaluate other atoms, apart from ^1^H that also have magnetic moment such as ^31^P, ^13^C, ^23^Na, and ^87^Rb [52]. The nuclei most investigated in human cardiac MRS is ^31^P, where a normal spectrum is composed by six phosphorus peaks including ATP (three peaks: γ, α, and β), phosphocreatine (PCr), phosphodiesters (PDE), and 2,3-diphosphoglycerate (2,3-PDG) [108] (Fig. 7). From the ^31^P spectrum, it is possible to calculate the PCr-to-ATP ratio which reflects the index of the energetic state of the heart [3]. In the context of ischemic heart disease, Weiss and collaborators demonstrated that in a cohort of 16 patients with coronary artery disease, during handgrip exercise, there was a transient imbalance between the oxygen supplied and required by the myocardium, which was reflected by a decreased ATP/PCr ratio measured by ^31^P-MRS [153]. ATP/PCR ratio returned to normal after recovery. This transient effect was not detected in healthy volunteers and nonischemic patients [153]. In addition, several approaches to evaluate the efficacy of treatment interventions after ischemia have been tested [28]; however, large-scale trials to investigate long-term effects are needed.

^1^H-MRS is more widely available and has significantly more sensitivity and, therefore, more realistic potential than ^31^P-MRS to become a clinical tool. However, the information obtained using ^1^H-MRS differs from the information obtained by ^31^P-MRS. ^1^H-MRS allows the measurement of important metabolites such as creatine, lactate, carnitine, deoxymyoglobin, and cardiac lipids. The measurement of cardiac lipids provides information about the accumulation of triglycerides that are associated with impaired myocardial contractility [108]. ^23^Na-MRS, ^13^C-MRS, and ^87^Rb-MRS have been very little explored, mainly due to their very low sensitivity; however, ^23^Na signal has been correlated with acute necrosis and chronic myocardial scarring, therefore, a potential method to evaluate cardiac viability without the use of contrast agents [108]. ^13^C has very limited application in the myocardium; however, some studies revealed the applicability of this spectrum to evaluate metabolites from the Kreb’s cycle, β-oxidation of fatty acids and pyruvate flux. Finally, ^87^Rb is an analogue of K^+^, so it is believed that this spectrum can provide valuable information about Na^+^/K^+^ ATPase pumps [108]. One of the main advantages of MRS is the possible combination of the spectrums from different atoms that would provide a full characterisation of the myocardial state in coronary artery disease.

Cardiac MRS is a promising technique that could be used as a prognostic tool in the future. However, ^31^P-MRS is limited by its low spatial and temporal resolution and the low sensitivity of ^31^P (6.6% of ^1^H sensitivity). Technological advances using higher field strengths (> 3 T) have improved temporal and spatial resolutions and the signal-to-noise; however, more advanced coil design, well-defined protocols, and sequence development are required to translate this method into clinical practice.

## Hybrid PET/MR imaging

As we have stated throughout this review, both PET and MRI have been successfully in providing data for diagnosis, prognosis, and monitoring myocardial changes after myocardial infarction. Hybrid systems like PET/CT or SPECT/CT have already demonstrated their important clinical value. PET/MRI systems have entered the market recently, and allow the acquisition of the PET data simultaneously or sequentially to MRI data. It has been presented as a possible alternative to PET/CT due to the lower radiation exposure and the improved cardiac and respiratory motion compensation. However, one of the common disadvantages of hybrid systems and in particular PET/MRI is the increased complexity of the workflow, due to the higher complexity of the MRI compared to CT. However, there are several advantages of using MRI compared to CT, such as improved tissue characterization, tissue perfusion, diffusion, T1, T2, spectroscopic data, and motion estimation.

**Fig. 8:**
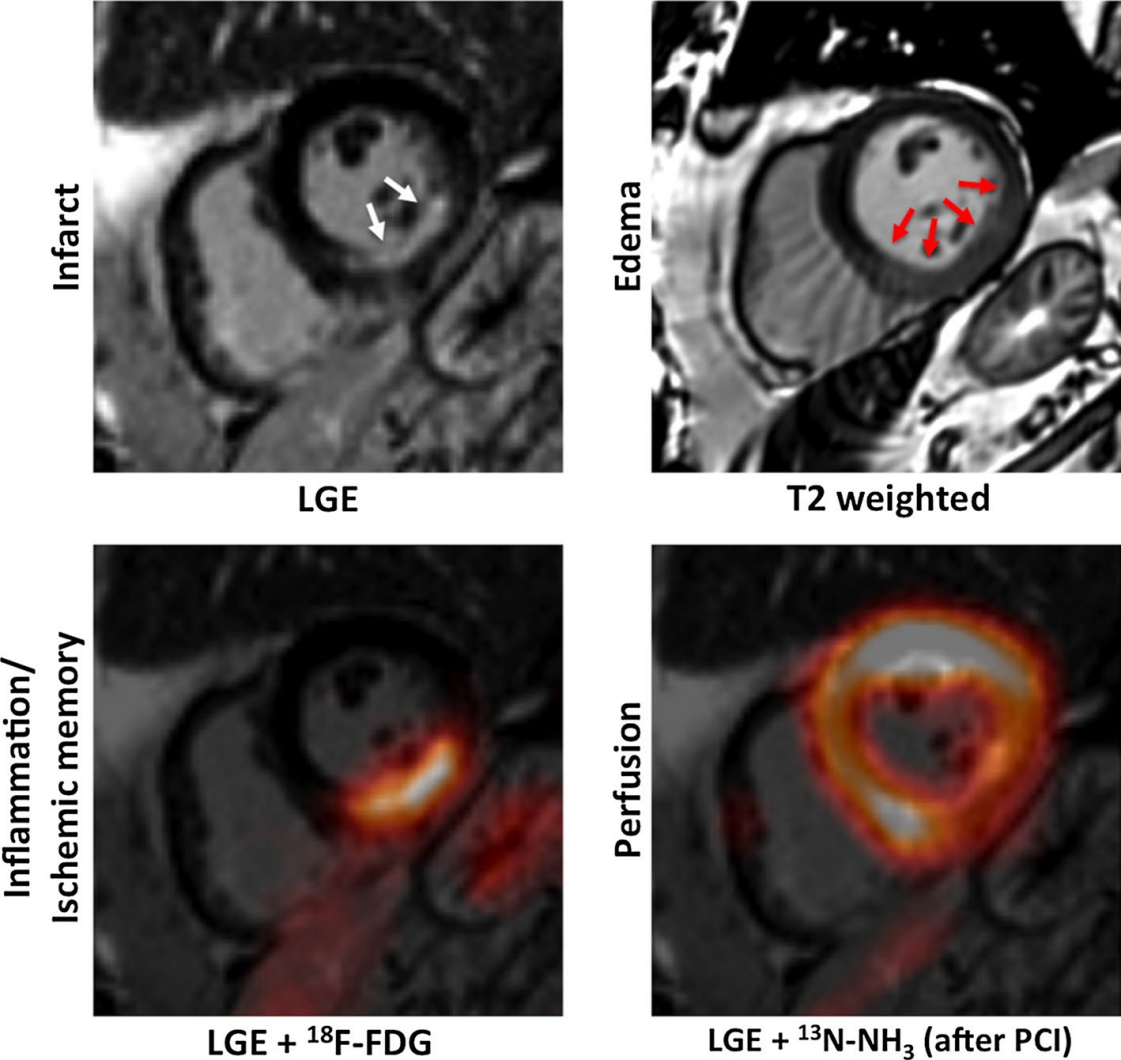
Multimodal characterization of the myocardial tissue after AMI using PET/MRI. Short-axis images of a patient who was imaged shortly after acute MI using simultaneous ^18^F-FDG and ^13^N-NH_3_ PET/MRI. Myocardial scarring can be imaged using LGE MRI (left column, top; white arrows pointing at subendocardial non-transmural infarction). The area of myocardial infarction is exceeded by the myocardial oedema imaged using T2-weighted sequences (right column, top; red arrows). Using fasting-heparin ^18^F-FDG-PET/MRI, the area of post-ischemic inflammation or ischemic memory can be assessed. After revascularization by percutaneous coronary intervention (PCI), only a slightly reduced perfusion of the inferior wall was observed in this patient [113]

Myocardial tissue characterization is usually performed using ^18^F-FDG-PET. However, the myocardial uptake may be reduced in diabetic patients resulting in poor image quality together with the low spatial resolution of PET not being enough to assess the distribution of the tracer through the myocardium [113] (Fig. 8). LGE MRI after administration of a Gd-contrast agent is an alternative and has become the standard of reference for viability assessment [68, 69]. There are only few small studies, where the feasibility of ^18^F-FDG-PET together with LGE MRI has been successfully tested [99]. Moreover, the use of ^18^F-NaF-PET/MRI has been successfully validated this year to detect myocardial scar in a small cohort of STEMI patients [87]. Our group has recently developed a simultaneous CMRA—FDG-PET protocol, whereby respiratory motion is estimated from the CMRA acquisition and used to correct both the MR and PET attenuation and emission data [96]. These examples show the feasibility of PET/MRI to assess myocardial changes after MI. However, this new technology is at the beginning of its development and technological advances and other challenges related to the complexity need to be evaluated. In addition, larger clinical validations in different pathologies are required to present PET/MRI as a real clinical alternative.

## Diffusion tractography

**Fig. 9:**
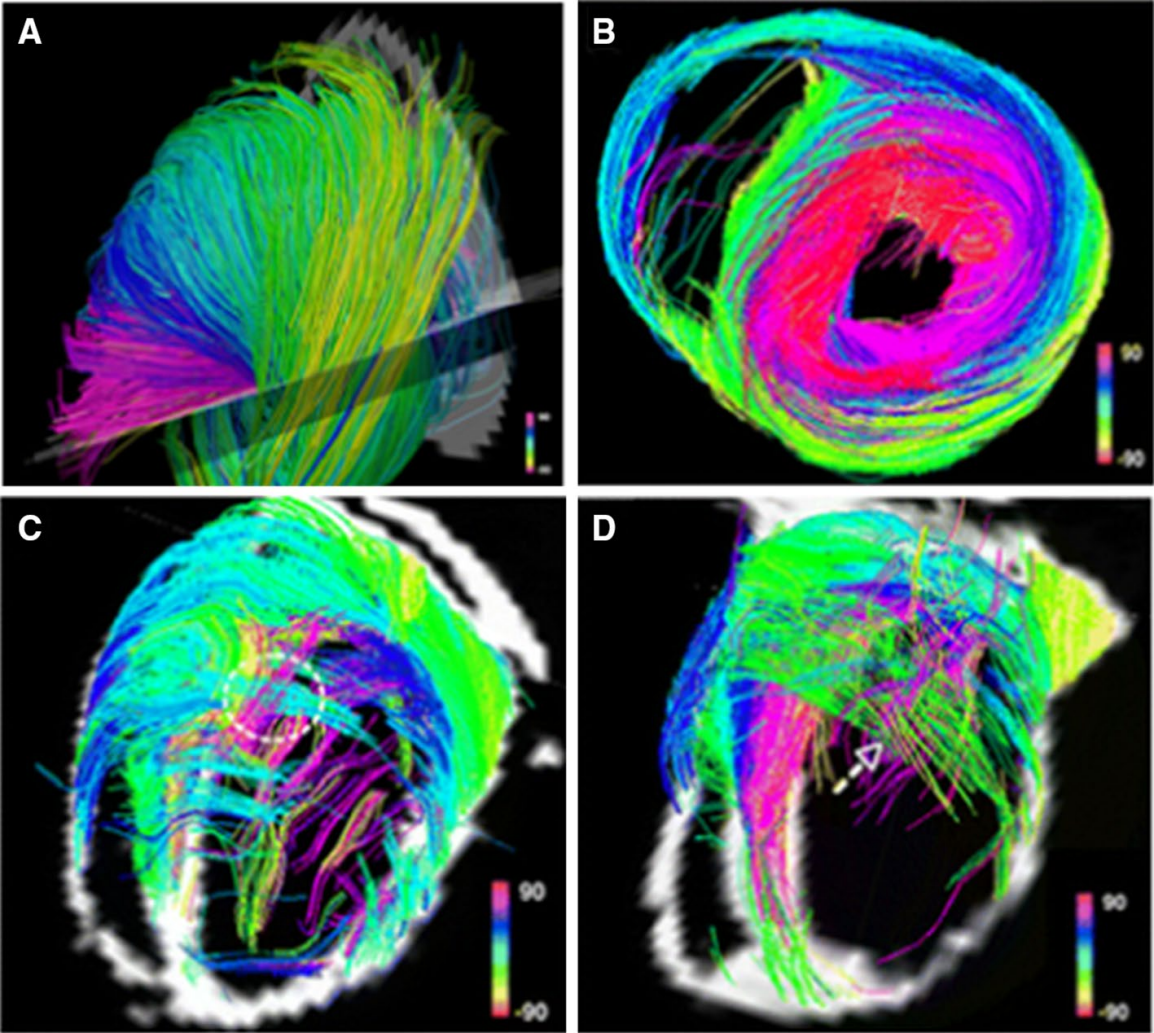
Diffusion tractography in a healthy rat heart (**a**, **b**). Two infarcted rat hearts show severe distortion of myofibre architecture (**c**, **d**) [125, 129]

Diffusion MRI tractography and its use in cardiac imaging are an active area of research. Diffusion tractography has already been well described in imaging of the matter tracts of the central nervous system [76]. Tractography consists of imaging the direction of particular fibres of interest, producing a diffusion tensor (a vector made up of numerous eigenvalues) for each voxel within an image and this helps determine its direction. The computing system determines the main direction followed by various voxels and draws a pathway following the main direction of the diffusion tensor. Diffusion tractography can be useful in cardiac imaging to evaluate the architecture of the myofibers within the heart (Fig. 9). It has been shown that in healthy hearts, the myofibre architecture is smooth and constant, whereas in infarcted hearts, there is a severe disruption in the architecture [125, 129].

## Discussion

Molecular cardiac imaging is a relatively new and exciting approach with a large scope for future work and improvements before its introduction into clinical practice. This review has focused on molecular imaging of post-MI cardiac remodelling which subsequently may lead to heart failure. These techniques allow visualisation of molecular pathways that occur during remodelling, allowing serial imaging showing disease progression and therapeutic response. Ultimately, this technology could be used by clinicians for prognostic purposes and for individual medication tailoring.

Although the current tracers have been successfully validated to image specific targets, they can always be improved. One of the chemical engineering challenges may be to achieve better sensitivity and stability of the current tracers and consequently superior images. Likewise, new tracers can be developed to broaden the library of tracers available, allowing the evaluation of different molecular pathways, and, therefore, increase our knowledge to provide a more personalised evaluation and treatment of patients. Some examples of possible targets that may have an important impact on cardiac imaging are pH, tissue oxygenation, troponin, creatinine kinase, and specific cell populations such as inflammatory or reparative monocyte/macrophages. However, the tracers discussed are very specific to their target and have proven to be successful at visualising their targets, and perhaps, at this stage, we should focus on these tracers to pursue translation to clinical practice as opposed to researching new potential tracers.

There is extensive literature on how bringing this research into clinical practice is an expensive and time-consuming task [62, 83, 123]. Many of these studies have been conducted on mice and multiple authors have described the different behaviour of the tracers between mice, pigs, and humans, especially in patients with co-morbidities and more complex cellular mechanisms [12, 62]. However, recently, we have seen human trials in many of the imaging techniques described. Although initial pilot studies, they have so far shown tracers to be safe in humans and to successfully visualise their targets. Clearly, more human studies are required to evaluate the safety and viability of these tracers, but translating this new technology into clinical practice is becoming more realistic than first thought. Once these tracers have been deemed safe for use in humans, large multi-centre clinical trials will be required to ensure they can successfully image their targets, provide prognostic information to patients and offer evidence to clinicians to individually tailor medication to the patient. Imaging techniques will only be considered for clinical practice if they are deemed safe, robust and have a clinically relevant outcome that will aid or change the management of a patient. We are confident that these techniques will make it to the bedside in the future and will enable clinicians to monitor disease progression and therapeutic response.

Furthermore, these techniques could be used as an adjunct to novel molecular therapeutics. Anti-inflammatory and pro-angiogenic or other molecular therapies have recently been studied [5, 65, 111]. The imaging techniques described in this review can act as guidance for these therapies both in experimental studies and clinically if these therapies make it to the bedside.

Efficient healthcare spending is vitally important in today’s climate and the question of cost-efficiency of this new technology must be asked. However, as imaging systems become more advanced and scan acquisition times decrease so does the price of running a single scan, and given the significant cost of heart failure on healthcare systems if these techniques contribute to a decrease in morbidity and mortality then perhaps overall spending would be reduced. Naturally, as imaging systems continue to improve so will the imaging techniques described in this review. As PET/MRI hybrid systems are a recent addition to the market, this will also become an active area of research in this field as these two individual modalities make up the majority of this field.

This field is in an exciting phase, given the recent human trials. Further evaluation and clinical trials need to be implemented. Ultimately, more work needs to be done in this area before molecular cardiac imaging becomes part of the clinician’s toolbox; however, large steps have been taken recently and clinical translation is becoming ever more promising.

## Conclusion

The main clinical experience with cardiac molecular imaging is with nuclear imaging due to the availability of tracers, their high sensitivity, and low risk. New molecular imaging techniques have been proposed and studied within both nuclear imaging and MRI. SPECT, PET, and MRI have the ability to image different cardiac processes, providing an extensive, non-invasive examination of the infarct process, and subsequent healing. Early human trials have shown promising results, and while significant challenges remain, these advances have shown potential advantages and may lead to improved, more individualised patient management. Large, multi-centre clinical trials are needed for safety evaluation of tracers in addition to diagnostic and prognostic value confirmation.
